# On the Spectrum of the Local ${\mathbb {P}}^2$ Mirror Curve

**DOI:** 10.1007/s00023-020-00960-y

**Published:** 2020-09-29

**Authors:** Rinat Kashaev, Sergey Sergeev

**Affiliations:** 1 Section de Mathématiques, Université de Genève, 2-4 rue du Lièvre, Case Postale 64, 1211 Genève 4, Switzerland; 2 Department of Theoretical Physics, Research School of Physics and Engineering, Australian National University, Canberra, ACT 0200 Australia

**Keywords:** Primary 39A13, Secondary 33E30

## Abstract

We address the spectral problem of the formally normal quantum mechanical operator associated with the quantised mirror curve of the toric (almost) del Pezzo Calabi–Yau threefold called local ${\mathbb {P}}^2$ in the case of complex values of Planck’s constant. We show that the problem can be approached in terms of the Bethe ansatz-type highly transcendental equations.

## Introduction

The recent progress in topological string theory reveals connections between spectral theory, integrable systems and local mirror symmetry. The results on linkings of some quantum mechanical spectral problems with integrable systems and conformal field theory [4, 6], together with the relation of topological strings in toric Calabi–Yau manifolds to integrable systems [1, 2, 10–13, 17, 19, 20], have lead to the conjecture on the topological string/spectral theory (TS/ST) correspondence [5, 8]. In many cases, quantisation of mirror curves produces trace class quantum mechanical operators, and according to the TS/ST correspondence, their spectra seem to contain a great deal of information of the enumerative geometry of the underlying Calabi–Yau manifold; see [16] for a review and references therein.

In the case of toric (almost) del Pezzo Calabi–Yau threefold known as local ${\mathbb {P}}^2$, the corresponding operator is of the form

1 $$\begin{aligned} \varvec{O}_{\mathbb {P}^2}=\varvec{u}+\varvec{v}+e^{i\hbar /2}\varvec{v}^{-1}\varvec{u}^{-1} \end{aligned}$$

with invertible positive self-adjoint operators $\varvec{u}$ and $\varvec{v}$ such that

2 $$\begin{aligned} \varvec{u}\varvec{v}=e^{i\hbar }\varvec{v}\varvec{u}, \end{aligned}$$

where $\hbar$ is a real strictly positive parameter. With various levels of generality, the spectral problem for similar operators has been addressed in [15] from the perspective of exact WKB approximation, in [9, 18] using a matrix integral representation of the eigenfunctions and in [3, 14, 22] from the standpoint of quantum integrable systems.

In this paper, following the approach of [14, 22], we consider the so-called strongly coupled regime for the operator (1) with invertible normal operators $\varvec{u}$ and $\varvec{v}$ satisfying the Heisenberg–Weyl relation (2) where

3 $$\begin{aligned} \hbar =2\pi e^{i2\theta },\quad \theta \in ]0,\pi /2[. \end{aligned}$$

In this case, we show that a realisation of the operator (1) in the Hilbert space $L^2(\mathbb {R})$ is formally normal so that its spectral problem is still well defined provided the operator admits a normal extension. Our main result is Theorem 2 which describes a set of solutions of our spectral problem in terms of generalised the Bethe ansatz-type transcendental equations.

### Outline

In Sect. 2, we introduce the setup, fix notation and conventions and introduce some objects and constructions that will be used subsequently in the rest of the paper. In Sect. 3, we specify our Hamiltonian as an unbounded operator in $L^2(\mathbb {R})$, show that it is formally normal, rewrite the corresponding spectral problem as a pair of functional difference equations and discuss a symmetry of the system called F-duality which corresponds to Faddeev’s modularity in integrable systems. In Sect. 4, we describe the possible F-dual asymptotics of solutions of the problem. In Sect. 5, we describe formal power series solutions for each asymptotic behaviour at infinity and establish their convergence properties. In Sect. 6, we develop an analytic tool for the realisation of the formal power series solutions with zero radius of convergence as asymptotic expansions of analytic solutions. This tool is based on the important technical Theorem 1. Section 7 is devoted to explicit constructions of analytic solutions. The specific vector spaces of analytic functions on the punctured complex plane $\mathbb {C}_{\ne 0}$ specified by linear difference equations play the central role here. In Sect. 8, we formulate and prove Theorem 2, the main result of the paper.

## Definitions and Notation

### Heisenberg Operators

Let $\varvec{x}$ and $\varvec{p}$ be normalised self-adjoint quantum mechanical *Heisenberg operators* defined by their realisation in the “position representation”

4 $$\begin{aligned} \langle x|\varvec{x}=x \langle x|,\quad \langle x|\varvec{p}=\frac{1}{2\pi i}\frac{\partial }{\partial x}\langle x| \end{aligned}$$

in the complex Hilbert space $L^2(\mathbb {R})$ of square integrable functions with respect to the standard Lebesgue measure. Here, we use Dirac’s bra–ket notation so that for any $\psi \in L^2(\mathbb {R})$ we write $\psi (x)=\langle x|\psi \rangle ,$

5 $$\begin{aligned} \langle x|\varvec{x}|\psi \rangle =x\langle x|\psi \rangle \end{aligned}$$

if $\psi$ is in the domain of $\varvec{x}$, and

6 $$\begin{aligned} \langle x|\varvec{p}|\psi \rangle =\frac{1}{2\pi i}\frac{\partial }{\partial x}\langle x|\psi \rangle \end{aligned}$$

if $\psi$ is in the domain of $\varvec{p}$. One can easily verify the Heisenberg commutation relation

7 $$\begin{aligned}{}[\varvec{p},\varvec{x}]{:}=\varvec{p}\varvec{x}-\varvec{x}\varvec{p}=\frac{1}{2\pi i}, \end{aligned}$$

where in the right-hand side the scalar is assumed to be the multiple of the identity operator.

### Heisenberg–Weyl Operators

We fix $\theta \in ]0,\pi /2[$, denote

8 $$\begin{aligned} \mathsf {b}{:}=e^{i\theta },\ \bar{\mathsf {b}}{:}=\frac{1}{\mathsf {b}},\ {\mathsf {q}}{:}=e^{\pi i \mathsf {b}^2},\ \bar{\mathsf {q}}{:}=e^{-\pi i {\bar{\mathsf {b}}}^{2}},\ c_\mathsf {b}{:}=\frac{i}{2}(\mathsf {b}+{\bar{\mathsf {b}}})=i\cos \theta \end{aligned}$$

and define the normal *Heisenberg–Weyl operators*

9 $$\begin{aligned} \varvec{u}{:}=e^{2\pi \mathsf {b}\varvec{x}},\quad \varvec{v}{:}=e^{2\pi \mathsf {b}\varvec{p}} \end{aligned}$$

together with their Hermitian conjugates

10 $$\begin{aligned} \varvec{u}^\dagger =e^{2\pi {\bar{\mathsf {b}}}\varvec{x}},\quad \varvec{v}^\dagger =e^{2\pi {\bar{\mathsf {b}}}\varvec{p}}. \end{aligned}$$

These operators satisfy (formally) the commutation relations

11 $$\begin{aligned} \varvec{u}\varvec{v}=q^2\varvec{v}\varvec{u},\quad \varvec{u}\varvec{v}^\dagger =\varvec{v}^\dagger \varvec{u}\end{aligned}$$

so that if $\varvec{a}$ and $\varvec{b}$ are arbitrary elements of the algebra generated by $\varvec{u}$ and $\varvec{v}$, then formally one has $\varvec{a}\varvec{b}^\dagger =\varvec{b}^\dagger \varvec{a}$.

### A Sequence of Polynomials

To any $n\in \mathbb {Z}_{\ge 0}$, we associate a polynomial $P_n=P_n(q,E)\in \mathbb {Z}[q,q^{-1}][E]$ of degree *n* in *E* defined by the following recurrence equation

12 $$\begin{aligned} P_{n+1}=EP_{n}+(q^n-q^{-n})(q^{n-1}-q^{1-n})P_{n-2},\quad P_0=1. \end{aligned}$$

Notice the symmetry $P_n(q,E)=P_n(1/q,E)$. Denoting $q_n{:}=q^n-q^{-n}$, the few first polynomials read as follows:

13 $$\begin{aligned} P_0= & {} 1,\quad P_1=E, \quad P_2=E^2,\quad P_3=E^3+q_1q_2,\quad P_4=E^4+\frac{q_2^3}{q_1}E,\nonumber \\ P_5= & {} E^5+\frac{(q_1^2+q_3^2)q_2}{q_1}E^2,\quad P_6=E^6+\frac{(q_2^2+5)q_2^3}{q_1}E^3+q_1q_2q_4q_5,\quad \dots \end{aligned}$$

Among the properties of these polynomials, one can show that $P_n(q,0)=0$ unless $n\equiv 0\pmod 3$ and

14 $$\begin{aligned} P_{3m}(q,0)=q^{-3m^2}(q^2;q^6)_m(q^4;q^6)_m,\quad \forall m\in {\mathbb {Z}}_{\ge 0}, \end{aligned}$$

where we use the standard *q*-*Pochhammer symbol*

15 $$\begin{aligned} (x;q)_n{:}=\prod _{k=0}^{n-1}(1-xq^k), \quad (x,y,\dots ;q)_n{:}=(x;q)_n(y;q)_n\cdots \end{aligned}$$

One can also show that

16 $$\begin{aligned} P_{n}=\sum _{0\le m\le n/3}E^{n-3m}p_{n,m},\quad p_{n,m}=\sum _{|k|\le m}q^{2nk}\alpha _{k,m}(n), \end{aligned}$$

where $\alpha _{k,m}(x)$ are polynomials in *x* of degree $m-|k|$ satisfying the recurrence relations

17 $$\begin{aligned}&q^{6k}\alpha _{k,m+1}(x+3)-q^{4k}\alpha _{k,m+1}(x+2)\nonumber \\&\quad =q^3\alpha _{k-1,m}(x)+q^{-3}\alpha _{k+1,m}(x)-(q+q^{-1})\alpha _{k,m}(x). \end{aligned}$$

Furthermore, the leading asymptotics of $P_n$ at large *n* is given by the formula

18 $$\begin{aligned} P_n(q,E)\vert _{n\rightarrow \infty }\sim q^{-n^2/3}. \end{aligned}$$

It will be of particular interest for us the following two generating series for these polynomials:

19 $$\begin{aligned} \phi _{q,E}(z){:}=\sum _{n=0}^\infty \frac{P_n(q,E)}{(q^{-2};q^{-2})_n}z^n \end{aligned}$$

and

20 $$\begin{aligned} \psi _{q,E}(z){:}=\sum _{n=0}^\infty \frac{P_n(q,E)q^{(1-n)n/2}}{(q^{-2};q^{-2})_n}z^n=\psi _{1/q,E}(-z). \end{aligned}$$

The asymptotics (18) implies that for any $E\in \mathbb {C}$, the radius of convergence is infinite for the series (20), while for the series (19) it is infinite if $|q|<1$ and zero if $|q|>1$.

### Vector Spaces $F_{p,c}$, $G_{p,c}$ and $T^m_{p,r}$

We let $H(\mathbb {C}_{\ne 0})$ denote the complex vector space of holomorphic functions $f:\mathbb {C}_{\ne 0}\rightarrow \mathbb {C}$ and

21 $$\begin{aligned} U(f){:}=\mathbb {C}_{\ne 0}\setminus f^{-1}(0),\quad f\in H(\mathbb {C}_{\ne 0}). \end{aligned}$$

Let $c\in \mathbb {C}$, $p,r\in \mathbb {C}_{\ne 0}$ and $m\in \mathbb {Z}$. We define the following vector subspaces of $H(\mathbb {C}_{\ne 0})$:

22 $$\begin{aligned}&F_ {p,c}{:}=\left\{ f\in H(\mathbb {C}_{\ne 0})\mid f(z/p^2)+(zp)^3f(zp^2)=(1-cz)f(z)\right\} , \end{aligned}$$

23 $$\begin{aligned}&G_ {p,c}{:}=\left\{ f\in H(\mathbb {C}_{\ne 0})\mid f(zp)-f(z/p)=z(z^2+c)f(z)\right\} \end{aligned}$$

and

24 $$\begin{aligned} T^m_ {p,r}{:}=\left\{ f\in H(\mathbb {C}_{\ne 0})\mid rz^mf(zp)=f(z)\right\} . \end{aligned}$$

Elements of $T^m_ {p,r}$ will be called *theta functions* of *order* *m*. For any $p\in \mathbb {C}_{\ne 0}$ such that $|p|<1$, one specific theta function is defined by the series

25 $$\begin{aligned} \vartheta (z;p){:}=\sum _{n\in \mathbb {Z}}p^{(n-1)n/2}(-z)^n=(z,p/z,p;p)_\infty \end{aligned}$$

with the defining properties

26 $$\begin{aligned} \vartheta (1;p)=0,\quad \vartheta (pz;p)=\vartheta (z^{-1};p)=-\frac{1}{z}\vartheta (z;p) \end{aligned}$$

which imply that $\vartheta (\cdot ;p)\in T^1_{p,-1}$. Note also the *modularity property*

27 $$\begin{aligned} \vartheta (e^{2\pi \mathsf {b}x};\mathsf {q}^2)=\vartheta (e^{2\pi {\bar{\mathsf {b}}} x};\bar{\mathsf {q}}^2)e^{\pi i(x+\sin \theta )^2-i\theta +\pi i/4}, \end{aligned}$$

where we use notation (8).

#### Remark 1

Let $f\in T^m_{p,r}$ and $z\in U(f)$ (respectively, $z\not \in U(f)$). Then, one has $zp^{\mathbb {Z}}\subset U(f)$ (respectively, $zp^{\mathbb {Z}}\cap U(f)=\{\}$).

#### Remark 2

By expanding into Laurent series, one easily checks that the dimensions of $$F_{p,c}$$ and $$ G_{p,c}$$ are at most 3. On the other hand, the recurrence relation (12) implies that $$\psi _{p,c}\in G_{p,c}$$ if $$|p|\ne 1$$ and $$\phi _{p,c}\in F_{p,c}$$ if $$|p|<1$$ so that

28 $$\begin{aligned} |p|\ne 1\Rightarrow \dim G_{p,c}\ge 1 \end{aligned}$$

and

29 $$\begin{aligned} |p|<1\Rightarrow \dim F_{p,c}\ge 1. \end{aligned}$$

The elements $\phi _{p,c}$ and $\psi _{p,c}$ will be called *regular elements* of the corresponding vector spaces.

#### Remark 3

By expanding into Laurent series, it is easily verified that

30 $$\begin{aligned} \dim T^m_{p,r}\le |m|\ \text { if }\ m\ne 0 \end{aligned}$$

with the equality if $|p|^m<1$. In particular, for $|p|<1$, $\dim T^1_{p,r}=1$ with the theta function $\vartheta (-r z;p)$ being a basis element.

#### Remark 4

One has the identifications

31 $$\begin{aligned} T^0_{1,1}= & {} H(\mathbb {C}_{\ne 0}), \end{aligned}$$

32 $$\begin{aligned} T^0_{p,1}= & {} \mathbb {C}\ \text { if }\ 1\not \in p^{\mathbb {Z}_{\ne 0}}, \end{aligned}$$

and the inclusions

33 $$\begin{aligned} \begin{aligned} T^m_{p,r}\subset T^{mn}_{p^n,r^np^{mn(n-1)/2}},\quad \forall m,n\in \mathbb {Z},\ \forall p,r\in \mathbb {C}_{\ne 0}, \end{aligned} \end{aligned}$$

which for $n=-1$ become equalities

34 $$\begin{aligned} T^m_{p,r}= T^{-m}_{p^{-1},r^{-1}p^{m}},\quad \forall m\in \mathbb {Z},\ \forall p,r\in \mathbb {C}_{\ne 0}. \end{aligned}$$

#### Remark 5

The multiplication of functions induces a linear map

35 $$\begin{aligned} T^m_{p,r}\otimes T^n_{p,s}\rightarrow T^{m+n}_{p,rs},\quad \forall m,n\in \mathbb {Z},\ \forall p,r,s\in \mathbb {C}_{\ne 0}. \end{aligned}$$

For example, assuming $|p|<1$, the product identity

36 $$\begin{aligned} \vartheta (z;p)\vartheta (-z;p)=\vartheta (p;p^2)\vartheta (z^2;p^2) \end{aligned}$$

illustrates the special case $T^1_{p,-1}\otimes T^1_{p,1}\rightarrow T^{2}_{p,-1}$.

#### Remark 6

Assuming $1\not \in p^{\mathbb {Z}_{\ne 0}}$, let $g\in G_{p,c}$. Then, the even part of the product $g(z)g(-zp)$ is an element of the vector space $T^0_{p,1}=\mathbb {C}$. Thus, there exists a quadratic form $\omega :G_{p,c}\rightarrow \mathbb {C}$ such that

37 $$\begin{aligned} g(z)g(-zp)+g(-z)g(zp)=\omega (g),\quad \forall z\in \mathbb {C}_{\ne 0}. \end{aligned}$$

In particular, if $|p|\ne 1$, we have

38 $$\begin{aligned} \omega (\psi _{p,c})=2\psi _{p,c}(0)=2. \end{aligned}$$

## Formulation of the Spectral Problem

Let $\varvec{u}$ and $\varvec{v}$ be the normal Heisenberg–Weyl operators defined in (9). Then, the *Hamiltonian*

39 $$\begin{aligned}&\varvec{H}{:}=\varvec{u}+\varvec{v}+\mathsf {q}^{-1}\varvec{u}^{-1}\varvec{v}^{-1}=\varvec{u}+\varvec{v}+{\mathsf {q}}\varvec{v}^{-1}\varvec{u}^{-1}\nonumber \\&\quad = e^{2\pi \mathsf {b}\varvec{x}}+e^{2\pi \mathsf {b}\varvec{p}}+e^{-2\pi \mathsf {b}(\varvec{x}+\varvec{p})} \end{aligned}$$

with the standard domain for sums of operators

40 $$\begin{aligned} {\mathcal {D}}\big (\varvec{H}\big )={\mathcal {D}}\big (e^{2\pi \mathsf {b}\varvec{x}}\big ) \cap {\mathcal {D}}\big (e^{2\pi \mathsf {b}\varvec{p}}\big )\cap {\mathcal {D}}\big (e^{-2\pi \mathsf {b}(\varvec{x}+\varvec{p})}\big ) \end{aligned}$$

is expected to extend to a normal operator so that the spectral problem consisting in solving the system of *Schrödinger equations*

41 $$\begin{aligned} {\varvec{H}}|\Psi \rangle =E|\Psi \rangle ,\quad {\varvec{H}}^\dagger |\Psi \rangle =\bar{E}|\Psi \rangle \end{aligned}$$

in the Hilbert space $L^2(\mathbb {R})$ is well posed. Below we justify this expectation by showing that ${\varvec{H}}$ is at least formally normal.

### The formal Normality of ${\varvec{H}}$

Recall that a densely defined linear operator *T* with the domain ${\mathcal {D}}(T)$ is called *formally normal* if ${\mathcal {D}}(T)\subset {\mathcal {D}}(T^\dagger )$ and

42 $$\begin{aligned} \Vert Tx\Vert =\Vert T^\dagger x\Vert \quad \forall x\in {\mathcal {D}}(T). \end{aligned}$$

A formally normal operator *T* is called *normal* if ${\mathcal {D}}(T)= {\mathcal {D}}(T^\dagger )$; see, for example, [21]. The notion of a formally normal operator naturally generalises the notion of a symmetric operator when one extends the class of self-adjoint operators to the class of normal operators.

#### Lemma 1

Let $\{\varvec{a}_j\}_{j\in J}$ be a finite set of densely defined operators such that $\varvec{A}{:}=\sum _{j\in J}\varvec{a}_j$ is densely defined, and for any $j,k\in J$, the operator $\varvec{a}_j+\varvec{a}_k$ is formally normal. Then $\varvec{A}$ is formally normal as well.

#### Proof

The case with $j=k$ implies that the operator $\varvec{a}_j$ is formally normal for any $j\in J$. This immediately implies the inclusion of domains

43 $$\begin{aligned} {\mathcal {D}}(\varvec{A})=\bigcap _{j\in J}{\mathcal {D}}(\varvec{a}_j) \subset \bigcap _{j\in J}{\mathcal {D}}(\varvec{a}_j^\dagger ) ={\mathcal {D}}\Big (\sum _{j\in J}\varvec{a}_j^\dagger \Big ) \subset {\mathcal {D}}(\varvec{A}^\dagger ), \end{aligned}$$

where the last inclusion is due to the inclusion of operators^1^$\sum _{j\in J}\varvec{a}_j^\dagger \subset \varvec{A}^\dagger .$

For any $j,k\in J$, by taking into account the inclusion of operators $\varvec{a}_j^\dagger +\varvec{a}_k^\dagger \subset (\varvec{a}_j+\varvec{a}_k)^\dagger$ as well as the formal normality of $\varvec{a}_j$ and $\varvec{a}_k$, we have the following equivalent characterisation of the formal normality of $\varvec{a}_j+\varvec{a}_k$:

44 $$\begin{aligned} M_{j,k}(x)=-M_{k,j}(x)\quad \forall x\in {\mathcal {D}}(\varvec{a}_j+\varvec{a}_k), \end{aligned}$$

where

45 $$\begin{aligned} M_{j,k}(x){:}=\langle \varvec{a}_jx|\varvec{a}_kx\rangle -\langle \varvec{a}_j^\dagger x|\varvec{a}_k^\dagger x\rangle . \end{aligned}$$

Indeed, this is verified as follows:

46 $$\begin{aligned} 0= & {} \Vert (\varvec{a}_j+\varvec{a}_k)x\Vert ^2-\Vert (\varvec{a}_j+\varvec{a}_k)^\dagger x\Vert ^2=\Vert (\varvec{a}_j+\varvec{a}_k)x\Vert ^2-\Vert (\varvec{a}_j^\dagger +\varvec{a}_k^\dagger )x\Vert ^2\nonumber \\= & {} M_{j,k}(x)+M_{k,j}(x). \end{aligned}$$

Thus, for any $x\in {\mathcal {D}}({\varvec{A}})$, we obtain

47 $$\begin{aligned} \Vert {\varvec{A}}x\Vert ^2-\Vert {\varvec{A}}^\dagger x\Vert ^2= & {} \sum _{j,k\in J}M_{j,k}(x)=-\sum _{j,k\in J}M_{k,j}(x)=\Vert {\varvec{A}}^\dagger x\Vert ^2-\Vert {\varvec{A}}x\Vert ^2\nonumber \\ \Leftrightarrow \Vert {\varvec{A}}x\Vert= & {} \Vert {\varvec{A}}^\dagger x\Vert . \end{aligned}$$

$$\square $$

#### Lemma 2

Let $\mathsf {b}=e^{i\theta }$ with $\theta \in ]0,\frac{\pi }{2}[$. Then, the set of three operators

48 $$\begin{aligned} \{\varvec{a}_1{:}=e^{2\pi \mathsf {b}\varvec{x}},\ \varvec{a}_2{:}=e^{2\pi \mathsf {b}\varvec{p}},\ \varvec{a}_3{:}=e^{-2\pi \mathsf {b}(\varvec{x}+\varvec{p})}\} \end{aligned}$$

satisfies the assumptions of Lemma 1.

#### Proof

For any $j\in \{1,2,3\}$, the operator $\varvec{a}_j$ is obviously normal. Let us check that $\varvec{a}_1+\varvec{a}_2$ is formally normal.

By using notation (8), recall that Faddeev’s quantum dilogarithm

49 $$\begin{aligned} \Phi _{\mathsf {b}}(z)=\frac{(-{\mathsf {q}}e^{2\pi \mathsf {b}z};{\mathsf {q}}^2)_\infty }{(-\bar{\mathsf {q}}e^{2\pi \bar{\mathsf {b}}z};\bar{\mathsf {q}}^2)_\infty } \end{aligned}$$

is a meromorphic function of $z\in \mathbb {C}$ which satisfies the functional equation

50 $$\begin{aligned} \frac{ \Phi _{\mathsf {b}}(z-i\mathsf {b}/2)}{ \Phi _{\mathsf {b}}(z+i\mathsf {b}/2)}=1+e^{2\pi \mathsf {b}z} \end{aligned}$$

and the unitarity condition

51 $$\begin{aligned} \overline{ \Phi _{\mathsf {b}}(z)}\Phi _{\mathsf {b}}({\bar{z}})=1, \end{aligned}$$

and it has neither zeros nor poles in the strip $|\mathfrak {I}(z)|<\cos (\theta )$. By using these properties, we write

52 $$\begin{aligned} \varvec{a}_1+\varvec{a}_2= & {} e^{2\pi \mathsf {b}\varvec{x}}+e^{2\pi \mathsf {b}\varvec{p}}=e^{\pi \mathsf {b}\varvec{x}}(1+e^{2\pi \mathsf {b}(\varvec{p}-\varvec{x})})e^{\pi \mathsf {b}\varvec{x}}\nonumber \\= & {} e^{\pi \mathsf {b}\varvec{x}}\frac{\Phi _{\mathsf {b}}(\varvec{p}-\varvec{x}-i\mathsf {b}/2)}{\Phi _{\mathsf {b}}(\varvec{p}-\varvec{x}+i\mathsf {b}/2)}e^{\pi \mathsf {b}\varvec{x}} =Ue^{2\pi \mathsf {b}\varvec{x}}U^\dagger , \end{aligned}$$

where $U{:}=\Phi _{\mathsf {b}}(\varvec{p}-\varvec{x})$ is a unitary operator and we used the following equalities:

53 $$\begin{aligned} e^{\pi s \varvec{x}}\Phi _{\mathsf {b}}(\varvec{p}-\varvec{x}-i s/2)=\Phi _{\mathsf {b}}(\varvec{p}-\varvec{x}) e^{\pi s \varvec{x}}=Ue^{\pi s \varvec{x}},\quad s\in \{\mathsf {b},{\bar{\mathsf {b}}}\}, \end{aligned}$$

which hold true due to the fact that the complex shifts in the argument of $\Phi _{\mathsf {b}}(z)$ are within the strip free of zeros and poles:

54 $$\begin{aligned} |\mathfrak {I}(i\mathsf {b}/2)|=|\mathfrak {I}(i{\bar{\mathsf {b}}}/2)|=\frac{1}{2}\cos (\theta )<\cos (\theta ). \end{aligned}$$

Thus, $\varvec{a}_1+\varvec{a}_2$ admits a normal extension, and in particular, it is formally normal. The formal normality of $\varvec{a}_1+\varvec{a}_3$ and $\varvec{a}_2+\varvec{a}_3$ is verified similarly. $\square$

We conclude that Lemma 1 in conjuction with Lemma 2 implies that the Hamiltonian (39) is a formally normal operator.

### The Difference Equations and F-Duality

In the position representation (4), the spectral problem (41) is equivalent to the following system of functional difference equations

55 $$\begin{aligned} \Psi (x-i\mathsf {b})+\mathsf {q}^{-1}e^{-2\pi \mathsf {b}x}\Psi (x+i\mathsf {b})= & {} (E-e^{2\pi \mathsf {b}x})\Psi (x), \end{aligned}$$

56 $$\begin{aligned} \Psi (x-i\bar{\mathsf {b}})+\bar{\mathsf {q}}e^{-2\pi \bar{\mathsf {b}} x}\Psi (x+i\bar{\mathsf {b}})= & {} {} (\bar{E}-e^{2\pi {\bar{\mathsf {b}}} x})\Psi (x), \end{aligned}$$

where $\Psi (x){:}=\langle x|\Psi \rangle$. We are looking for a holomorphic function $\Psi :\mathbb {C}\rightarrow \mathbb {C}$ that solves the functional Eqs. (55), (56) and whose restriction to the real axis is square integrable.

Equations (55) and (56) are related to each other by the simultaneous substitutions

57 $$\begin{aligned} \mathsf {b}\leftrightarrow {\bar{\mathsf {b}}},\quad \mathsf {q}\leftrightarrow 1/\bar{\mathsf {q}},\quad E\leftrightarrow {\bar{E}} \end{aligned}$$

which correspond to the *Faddeev (modular) duality* [7] and which we abbreviate as *F-duality*. For this reason, in what follows, we will write only one equation (containing the variables *E* and ${\mathsf {q}}$), but implicitly there will always be a second accompanying equation. In constructing solutions, we will follow the *principle of F-duality* corresponding to the invariance of solutions under above substitutions. In this case, it will suffice to check only one equation as the other one will be satisfied automatically.

## F-dual Asymptotics at $x\rightarrow \pm \infty$

We start our analysis by addressing the problem of asymptotical behaviour of solutions of our spectral problem at large values of *x*. Following the principle of F-duality, we are looking for possible F-dual asymptotics.

### Proposition 1

Let $\Psi (x)$ be a solution of Eqs. (55) and (56) such that there exists a finite nonzero limit of $e^{\lambda x}\Psi (x-i\mathsf {b})/\Psi (x)$ when *x* tends either to $+\infty$ or to $-\infty$ for some $\lambda \in \mathbb {C}$. Then, one has the following possibilities for the F-dual asymptotic behaviour of $\Psi (x)$ at large *x*:

58 $$\begin{aligned}&\Psi (x)\vert _{x\rightarrow -\infty }\sim \psi _0(x){:}= e^{-\pi ix^2/2 -\pi ic_{\mathsf {b}}x}, \end{aligned}$$

59 $$\begin{aligned}&\Psi (x)\vert _{x\rightarrow +\infty }\sim \psi _1(x){:}= e^{\pi ix^2 +2\pi ic_{\mathsf {b}}x}, \end{aligned}$$

and

60 $$\begin{aligned} \Psi (x)\vert _{x\rightarrow +\infty }\sim \psi _2(x){:}= e^{-2\pi ix^2 +2\pi ic_{\mathsf {b}}x}. \end{aligned}$$

### Proof

Dividing (55) by $\Psi (x)$, and denoting $\rho _\lambda (x){:}=e^{\lambda x}\Psi (x-i\mathsf {b})/\Psi (x)$, we obtain a first-order nonlinear finite difference functional equation with exponentially growing or decaying coefficients

61 $$\begin{aligned} \rho _\lambda (x)+e^{(2x+i\mathsf {b})(\lambda -\pi \mathsf {b})}/\rho _\lambda (x+i\mathsf {b})=(E-e^{2\pi \mathsf {b}x})e^{x\lambda }. \end{aligned}$$

Let $\lambda \in \mathbb {C}$ be such that there exists a finite nonzero limit value

62 $$\begin{aligned} \mu (\lambda ){:}=\lim _{x\rightarrow \infty }\rho _\lambda (x)=\lim _{x\rightarrow \infty }\rho _\lambda (x+i\mathsf {b}), \end{aligned}$$

where $\infty$ means one of $\pm \infty$, and assume that there exists an F-dual solution of the finite difference functional equation

63 $$\begin{aligned} e^{\lambda x}f_\lambda (x-i\mathsf {b})/f_\lambda (x)=\mu (\lambda ). \end{aligned}$$

Then, the corresponding asymptotic behaviour of $\Psi (x)$ is given by $f_\lambda (x)$.

**The case** $x\rightarrow -\infty$. Choosing $\lambda =\pi \mathsf {b}$, we obtain

64 $$\begin{aligned} \lim _{x\rightarrow -\infty }\left( \rho _{\pi \mathsf {b}}(x)+\frac{1}{\rho _{\pi \mathsf {b}}(x+i\mathsf {b})} \right) =0. \end{aligned}$$

Thus, one has two possibilities for the limit value

65 $$\begin{aligned} \mu (\pi \mathsf {b})=\epsilon i,\quad \epsilon \in \{\pm 1\}. \end{aligned}$$

The finite difference functional equation (63) admits an F-dual solution of the form $f_{\pi \mathsf {b}}(x)=\psi _0(x)$ provided $\epsilon =-1$.

**The case** $x\rightarrow +\infty$. We can choose either $\lambda =-2\pi \mathsf {b}$ or $\lambda =4\pi \mathsf {b}$ with the limit values

66 $$\begin{aligned} \mu (-2\pi \mathsf {b})=-1,\quad \mu (4\pi \mathsf {b})=-{\mathsf {q}}^3. \end{aligned}$$

The corresponding F-dual solutions of (63) are given by $f_{-2\pi \mathsf {b}}(x)=\psi _1(x)$ and $f_{4\pi \mathsf {b}}(x)=\psi _2(x)$. $\square$

Our further goal is to try to solve the eigenvalue Eqs. (55) and (56) in three asymptotic regimes (58)–(60) by using the substitutions $\Psi (x)=\psi _i(x)\varphi _i(x)$, $i\in \{0,1,2\}$ and looking for functions $\varphi _i(x)$ having finite nonzero limiting values in the corresponding asymptotic limits.

## Solutions in Terms of Power Series

### The Case $\psi _2(x)$

Assume that the asymptotic behaviour (60) takes place. If we write $\Psi (x)=\psi _2(x)\varphi _2(x)$, then the eigenvalue equation (55) is converted into the equation

67 $$\begin{aligned} \varphi _2(x+i\mathsf {b})+e^{-6\pi \mathsf {b}x}{\mathsf {q}}^3\varphi _2(x-i\mathsf {b})=(1-Ee^{-2\pi \mathsf {b}x})\varphi _2(x) \end{aligned}$$

which we complement with the limit value condition

68 $$\begin{aligned} \lim _{x\rightarrow +\infty }\varphi _2(x)=1. \end{aligned}$$

Under the F-dual substitution

69 $$\begin{aligned} \varphi _2(x)=\chi _2(e^{2\pi \mathsf {b}x}){\bar{\chi }}_2(e^{2\pi \bar{\mathsf {b}} x}), \end{aligned}$$

(67) is reduced to the equation

70 $$\begin{aligned} \chi _2(z{\mathsf {q}}^2)+({\mathsf {q}}/z)^3\chi _2(z/{\mathsf {q}}^2) =(1-E/z)\chi _2(z) \end{aligned}$$

which we complement with the limit value condition

71 $$\begin{aligned} \lim _{z\rightarrow \infty }\chi _2(z)=1. \end{aligned}$$

It admits a power series solution

72 $$\begin{aligned} \chi _2(z)=\phi _{\mathsf {q},E}(1/z). \end{aligned}$$

The dual function ${\bar{\chi }}_2(z)$ is obtained by the substitutions ${\mathsf {q}}\mapsto 1/\bar{\mathsf {q}}$ and $E\mapsto {\bar{E}}$ with the result

73 $$\begin{aligned} {\bar{\chi }}_2(z)=\phi _{1/\bar{\mathsf {q}},\bar{E}}(1/z). \end{aligned}$$

Taking into account the remarks in the end of Sect. 2.3, we conclude that $\chi _2(z)$ is holomorphic in $\mathbb {C}_{\ne 0}$, while ${\bar{\chi }}_2(z)$ does not converge to any complex analytic function.

### The Case $\psi _1(x)$

Assume that the behaviour (59) takes place. If we write $\Psi (x)=\psi _1(x)\varphi _1 (x)$, then the eigenvalue equation (55) is converted into the equation

74 $$\begin{aligned} \varphi _1(x-i\mathsf {b})+e^{-6\pi \mathsf {b}x}\mathsf {q}^{-3}\varphi _1(x+i\mathsf {b})=(1-Ee^{-2\pi \mathsf {b}x})\varphi _1(x) \end{aligned}$$

which we complement with the limit value condition

75 $$\begin{aligned} \lim _{x\rightarrow +\infty }\varphi _1(x)=1. \end{aligned}$$

As in the previous case, we obtain a power series F-dual solution

76 $$\begin{aligned} \varphi _1(x)=\phi _{1/\mathsf {q},E}(e^{-2\pi \mathsf {b}x})\phi _{\bar{\mathsf {q}},{\bar{E}}}(e^{-2\pi {\bar{\mathsf {b}}} x}), \end{aligned}$$

where the radii of convergence of the series $\phi _{1/\mathsf {q},E}(z)$ and $\phi _{\bar{\mathsf {q}},{\bar{E}}}(z)$ are zero and infinity, respectively.

### The Case $\psi _0(x)$

The substitution $\Psi (x)=\psi _0(x)\varphi _0 (x)$ converts the eigenvalue equation (55) into the equation

77 $$\begin{aligned} \varphi _0 (x-i\mathsf {b})-\varphi _0 (x+i\mathsf {b})=i(E-e^{2\pi \mathsf {b}x})e^{\pi \mathsf {b}x}\varphi _0 (x) \end{aligned}$$

which we complement with the limit value condition

78 $$\begin{aligned} \lim _{x\rightarrow -\infty }\varphi _0(x)=1. \end{aligned}$$

Under the F-dual substitution

79 $$\begin{aligned} \varphi _0 (x)=\frac{1}{2}\sum _{\sigma ,\tau \in \{0,1\}}(-1)^{\sigma \tau }\chi _0((-1)^\sigma e^{\pi \mathsf {b}x}){\bar{\chi }}_0((-1)^\tau e^{\pi {\bar{\mathsf {b}}} x}), \end{aligned}$$

(77) is equivalent to the following functional equation on the function $\chi _0(z)$:

80 $$\begin{aligned} \chi _0 (z/\mathsf {q})-\chi _0 (z\mathsf {q})=i(E-z^2)z\chi _0(z) \end{aligned}$$

complemented with the initial value condition

81 $$\begin{aligned} \chi _0(0)=1. \end{aligned}$$

It admits a power series solution $\chi _0(z)=\psi _{\mathsf {q},E}(-iz)$ and its dual ${\bar{\chi }}_0(z)=\psi _{\bar{\mathsf {q}},{\bar{E}}}(iz)$ with infinite radii of convergence. Thus, in this case, we obtain an entire function $\Psi (x)$.

## Analytic Realisations of the Series $\phi _{1/\mathsf {q},E}(z)$

### First-Order Matrix Difference Equation

For any $f\in F_{\mathsf {q},E}$, we have a matrix equality

82 $$\begin{aligned} {\hat{f}}(z)=L(z){\hat{f}}(z\mathsf {q}^2),\quad {\hat{f}}(z){:}= \begin{pmatrix} f(\frac{z}{\mathsf {q}^2})\\ f(z) \end{pmatrix},\ L(z){:}= \begin{pmatrix} 1-Ez&{}\quad -z^3\mathsf {q}^3\\ 1&{}\quad 0 \end{pmatrix}. \end{aligned}$$

Defining

83 $$\begin{aligned} L_n(z){:}=L(z)L(z\mathsf {q}^2)\cdots L(z\mathsf {q}^{2n-2})=: \begin{pmatrix} a_n(z)&{}\quad b_n(z)\\ c_n(z)&{}\quad d_n(z) \end{pmatrix},\quad n\in {{\mathbb {Z}}}_{>0}, \end{aligned}$$

we have

84 $$\begin{aligned} L_{m+n}(z)=L_m(z)L_n(z\mathsf {q}^{2m}),\quad \forall (m,n)\in ({\mathbb {Z}}_{>0})^2, \end{aligned}$$

which, in particular, implies that

85 $$\begin{aligned} L_{n+1}(z)=L(z)L_n(z\mathsf {q}^2)=L_n(z)L(z\mathsf {q}^{2n}),\quad \forall n\in {\mathbb {Z}}_{>0}. \end{aligned}$$

Taking the limit $n\rightarrow \infty$ in (83), relations (85) imply that

86 $$\begin{aligned} L_\infty (z){:}=\lim _{n\rightarrow \infty }L_n(z)= \begin{pmatrix} \phi _{\mathsf {q},E}(z/\mathsf {q}^2)&{}0\\ \phi _{\mathsf {q},E}(z)&{}0 \end{pmatrix}, \end{aligned}$$

where $\phi _{\mathsf {q},E}$ is the regular element of $F_{\mathsf {q},E}$. As we have seen in Sect. 2.3, it can be presented as the everywhere absolutely convergent series (19).

### The Wronskian Pairing

We define a skew-symmetric bilinear *Wronskian pairing*

87 $$\begin{aligned}{}[\cdot ,\cdot ]:F_{\mathsf {q},E}\times F_{\mathsf {q},E}\rightarrow T^3_{\mathsf {q}^2,\mathsf {q}^3},\quad [f,g](z)= f(\frac{z}{\mathsf {q}^2})g(z)-g(\frac{z}{\mathsf {q}^2})f(z). \end{aligned}$$

#### Remark 7

One can show that $\dim F_{\mathsf {q},E}=3$. As the kernel of the Wronskian pairing $[\phi _{\mathsf {q},E},\cdot ]$ contains $\phi _{\mathsf {q},E}$, we conclude that $\dim [\phi _{\mathsf {q},E},F_{\mathsf {q},E}]\le 2$.

### Adjoint Functions

For any $f\in F_{\mathsf {q},E}$, we associate the *adjoint function*

88 $$\begin{aligned} {\tilde{f}}:U([\phi _{\mathsf {q},E},f])\rightarrow {\mathbb {C}} \end{aligned}$$

(see (21) for the definition of the domain) defined by the formula

89 $$\begin{aligned} {\tilde{f}}(z){:}{=}\frac{f(z)}{[\phi _{\mathsf {q},E},f](z)}. \end{aligned}$$

By construction, ${\tilde{f}}(z)$ solves the functional equation

90 $$\begin{aligned} {\tilde{f}}(z\mathsf {q}^2)+(z/\mathsf {q})^3\tilde{f}(z/\mathsf {q}^2)=(1-E z){\tilde{f}}(z) \end{aligned}$$

obtained from the equation underlying the vector space $F_{\mathsf {q},E}$ by the replacement of ${\mathsf {q}}$ by $1/\mathsf {q}$. This equation admits a formal power series solution $\phi _{1/\mathsf {q},E}(z)$ which has zero radius of convergence. The adjoint functions appear to be analytic substitutes for $\phi _{1/\mathsf {q},E}$ due to the following theorem.

#### Theorem 1

Let $f\in F_{\mathsf {q},E}$ be such that the adjoint function ${\tilde{f}}(z)$ is a non-trivial meromorphic function. Then

91 $$\begin{aligned} \lim _{n\rightarrow \infty }{\tilde{f}}(z\mathsf {q}^{2n})=1,\quad \forall z\in U([\phi _{\mathsf {q},E},f]), \end{aligned}$$

so that ${\tilde{f}}(z)$ admits an asymptotic expansion at small *z* in the form of the series $\phi _{1/\mathsf {q},E}(z)$.

#### Proof

The proof is based on the matrix recurrence (82). Indeed, the formula

92 $$\begin{aligned} \det (L_n(z))=z^{3n} {\mathsf {q}}^{3n^2} \end{aligned}$$

implies that $L_n(z)$ is invertible for any $z\ne 0$, and we can write

93 $$\begin{aligned} {\hat{f}}(z)=L_n(z){\hat{f}}(z{\mathsf {q}}^{2n})\Leftrightarrow \hat{f}(z{\mathsf {q}}^{2n})=(L_n(z))^{-1}{\hat{f}}(z) \end{aligned}$$

so that

94 $$\begin{aligned} f(z{\mathsf {q}}^{2n})=\frac{a_n(z)f(z)-c_n(z)f(z/{\mathsf {q}}^2)}{z^{3n}{\mathsf {q}}^{3n^2}}, \end{aligned}$$

and taking into account the equality

95 $$\begin{aligned}{}[\phi _{\mathsf {q},E},f](z\mathsf {q}^{2n})=\frac{[\phi _{\mathsf {q},E},f](z)}{z^{3n} \mathsf {q}^{3n^2}}, \end{aligned}$$

we obtain

96 $$\begin{aligned} {\tilde{f}}(z\mathsf {q}^{2n})=\frac{a_n(z) f(z)-c_n(z) f(z/\mathsf {q}^2)}{[\phi _{\mathsf {q},E}, f](z)} \end{aligned}$$

which implies (91) due to the formulae

97 $$\begin{aligned} \lim _{n\rightarrow \infty }a_n(z)=\phi _{\mathsf {q},E}(z/\mathsf {q}^2),\quad \lim _{n\rightarrow \infty }c_n(z)=\phi _{\mathsf {q},E}(z), \end{aligned}$$

see (86), and the definition of the Wronskian pairing in (87). $\square$

Our next task is to construct elements of $F_{\mathsf {q},E}$ with non-trivial adjoint functions.

## Construction of Elements in $F_{\mathsf {q},E}$

### The Vector Space $V_ {\mathsf {q},\alpha ,E}$

Let $\alpha \in \mathbb {C}_{\ne 0}$. We define a vector space

98 $$\begin{aligned} V_ {\mathsf {q},\alpha ,E}{:}{=}\left\{ f\in H(\mathbb {C}_{\ne 0})\mid \alpha zf\Big (\frac{z}{\mathsf {q}^2}\Big )+\frac{z^2\mathsf {q}}{\alpha }f(z\mathsf {q}^2) =(1-Ez)f(z)\right\} . \end{aligned}$$

#### Proposition 2

Let $g\in G_{\mathsf {q},E}$. Consider the linear map

99 $$\begin{aligned} A_{g}:H(\mathbb {C}_{\ne 0})\rightarrow H(\mathbb {C}_{\ne 0}),\quad A_g(f)(z)=P_+(fg)(1/\sqrt{-z}), \end{aligned}$$

where $P_+$ is the projection to the even part of a function:

100 $$\begin{aligned} P_+(f)(z){:}{=}(f(z)+f(-z))/2. \end{aligned}$$

Then $A_{g}(T^1_{\mathsf {q},-\alpha })\subset V_{\mathsf {q},\alpha ,E}$ and the restriction $A_{g}\vert _{T^1_{\mathsf {q},-\alpha }}$ is a linear isomorphism between $T^1_{\mathsf {q},-\alpha }$ and $V_{\mathsf {q},\alpha ,E}$ provided $\omega (g)\ne 0$ (see Remark 6).

#### Proof

Let $h\in T^1_{\mathsf {q},-\alpha }$ and $f{:}{=}A_g(h)$. Denoting $u{:}{=}-1/z^2$, we have

101 $$\begin{aligned} 2u\alpha f(u/{\mathsf {q}}^2)= & {} -z^{-2}\alpha h(z{\mathsf {q}})g(z{\mathsf {q}})+(z\mapsto -z)\nonumber \\= & {} h(z)z^{-3}(g(z/{\mathsf {q}})+z(z^2+E)g(z))+(z\mapsto -z)\nonumber \\= & {} (-h(z/{\mathsf {q}})(\alpha z/{\mathsf {q}})^{-1}z^{-3}g(z/{\mathsf {q}})\nonumber \\&+(z\mapsto -z))+(h(z)(1+E/z^{2})g(z)+(z\mapsto -z))\nonumber \\= & {} -u^2{\mathsf {q}}\alpha ^{-1} 2f(u{\mathsf {q}}^2)+(1-Eu)2f(u). \end{aligned}$$

Thus, $$f\in V_{\mathsf {q},\alpha ,E}$$.

Assuming $\omega (g)\ne 0$, we solve the equality $f=A_g(h)$ for *h* as follows:

102 $$\begin{aligned}&2f(u)g(z{\mathsf {q}})+z\alpha g(z)2f(u/{\mathsf {q}}^2)\nonumber \\&\quad =(h(z)+z\alpha h(z{\mathsf {q}}))g(z)g(z{\mathsf {q}})+h(-z)g(-z)g(z{\mathsf {q}})+z\alpha g(z)h(-z{\mathsf {q}})g(-z{\mathsf {q}})\nonumber \\&\quad = h(-z)(g(-z)g(z{\mathsf {q}})+g(z)g(-z{\mathsf {q}}))=h(-z)\omega (g). \end{aligned}$$

Thus, *h* is determined through *f*:

103 $$\begin{aligned} h(z)=(f(-1/z^2)g(-z\mathsf {q})-z\alpha g(-z)f(-1/(z\mathsf {q})^2))2/\omega (g). \end{aligned}$$

$$\square $$

#### Corollary 1

For any $\alpha \in \mathbb {C}_{\ne 0}$ and $E\in \mathbb {C}$, one has

104 $$\begin{aligned} \dim (V_ {\mathsf {q},\alpha ,E})=\dim (T^1_{\mathsf {q},-\alpha })=1. \end{aligned}$$

In particular, the function

105 $$\begin{aligned} \chi _{{\mathsf {q}},\alpha ,E}(z){:}{=}\vartheta (\alpha z;{\mathsf {q}})\psi _{{\mathsf {q}},E}(z)+(z\mapsto -z) \end{aligned}$$

determines a basis element in $V_ {{\mathsf {q}},\alpha ,E}$.

#### Proof

Indeed, $\psi _{{\mathsf {q}},E}$ is an element of $G_{{\mathsf {q}},E}$ with $\omega (\psi _{\mathsf {q},E})=2\ne 0$, while $\vartheta (\alpha z;{\mathsf {q}})$ determines a basis element in $T^1_{{\mathsf {q}},-\alpha }$. $\square$

#### Remark 8

In the proof of the second part of Proposition 2, we implicitly used an extension of the Wronskian pairing

106 $$\begin{aligned}&[\cdot ,\cdot ]:G_{{\mathsf {q}},E}\times V_{{\mathsf {q}},\alpha ,E}\rightarrow T^1_{{\mathsf {q}},\alpha },\nonumber \\&[g,f](z)= g(z{\mathsf {q}})f\Big (\frac{-1}{z^2}\Big )+z\alpha f\Big (\frac{-1}{z^2{\mathsf {q}}^2}\Big )g(z) \end{aligned}$$

and the identity

107 $$\begin{aligned}{}[g,A_g(h)](z)=h(-z)\omega (g)/2,\quad \forall (g,h,z)\in G_{{\mathsf {q}},E}\times T^1_{{\mathsf {q}},-\alpha }\times \mathbb {C}_{\ne 0}. \end{aligned}$$

#### Proposition 3

The multiplication of functions induces a linear map

108 $$\begin{aligned} V_{{\mathsf {q}},\alpha ,E}\otimes T^1_{{\mathsf {q}}^2,{\mathsf {q}}^2\alpha }\rightarrow F_{{\mathsf {q}},E}. \end{aligned}$$

#### Proof

Let $f\in V_{{\mathsf {q}},\alpha ,E}$, $g\in T^1_{{\mathsf {q}}^2,{\mathsf {q}}^2\alpha }$ and $h{:}{=}fg$. We have

109 $$\begin{aligned} h(z/{\mathsf {q}}^2)= & {} (1-Ez)f(z)g(z)-z^2{\mathsf {q}}\alpha ^{-1}f(z{\mathsf {q}}^2)g(z)\nonumber \\= & {} (1-Ez)h(z)-(z{\mathsf {q}})^3h(z{\mathsf {q}}^2). \end{aligned}$$

Thus, $$h\in F_{{\mathsf {q}},E}$$. $$\square $$

### Adjoint Functions Revisited

Let $f\in V_{{\mathsf {q}},\alpha ,E}$, $g\in T^1_{{\mathsf {q}}^2,\mathsf {q}^2\alpha }$. Then, the adjoint function of the product *fg* takes the form

110 $$\begin{aligned} \widetilde{fg}(z)=\frac{f(z)g(z)}{[\phi _{{\mathsf {q}},E},fg](z)}=\frac{f(z)}{[\phi _{{\mathsf {q}},E},f](z)}, \end{aligned}$$

where, in the last expression, by abuse of notation, we extend the Wronskian pairing to include the space $V_{{\mathsf {q}},\alpha ,E}$

111 $$\begin{aligned}{}[\cdot ,\cdot ]:F_{{\mathsf {q}},E}\times V_{{\mathsf {q}},\alpha ,E}\rightarrow T^2_{{\mathsf {q}}^2,{\mathsf {q}}/\alpha },\quad [e,f](z)=e(\frac{z}{{\mathsf {q}}^2})f(z)-\alpha z f(\frac{z}{{\mathsf {q}}^2}) e(z). \end{aligned}$$

The inclusions of vector spaces in (33) specified to $T^2_{{\mathsf {q}}^2,{\mathsf {q}}/\alpha }$ become

112 $$\begin{aligned} T^2_{{\mathsf {q}}^2,{\mathsf {q}}/\alpha }\subset T^{2n}_{{\mathsf {q}}^{2n},{\mathsf {q}}^{n^2}/\alpha ^n},\quad \forall n\in \mathbb {Z}, \end{aligned}$$

which imply that

113 $$\begin{aligned}{}[\phi _{{\mathsf {q}},E},f](z)=0\Rightarrow [\phi _{{\mathsf {q}},E},f](z{\mathsf {q}}^{2n})=0,\quad \forall n\in \mathbb {Z}, \end{aligned}$$

and one has the equalities

114 $$\begin{aligned} \check{f}(z{\mathsf {q}}^{2n})={\mathsf {q}}^{n^2}(\alpha {\mathsf {q}}z)^n{\check{f}}(z), \quad \forall (n,z)\in \mathbb {Z}\times [\phi _{{\mathsf {q}},E},f]^{-1}(0)\cap U(\phi _{{\mathsf {q}},E}), \end{aligned}$$

where

115 $$\begin{aligned} {\check{f}}{:}{=}f/\phi _{{\mathsf {q}},E}. \end{aligned}$$

By adjusting the normalisation of *f*, we can write an equality

116 $$\begin{aligned}{}[\phi _{{\mathsf {q}},E},f](z)=\vartheta (z/s;{\mathsf {q}}^2)\vartheta (zs{\mathsf {q}} /\alpha ;{\mathsf {q}}^2),\quad \forall z\in \mathbb {C}_{\ne 0}, \end{aligned}$$

where $s=s(\alpha ,{\mathsf {q}},E)$ is a fixed zero of $[\phi _{{\mathsf {q}},E},f]$. We conclude that

117 $$\begin{aligned}{}[\phi _{{\mathsf {q}},E},f](z)=0\Leftrightarrow [\phi _{{\mathsf {q}},E},f](\frac{\alpha }{{\mathsf {q}}z})=0. \end{aligned}$$

## Solution of the Schrödinger Equations

Define the notation

118 $$\begin{aligned} p_x{:}{=}e^{-2\pi \mathsf {b}x},\quad {\bar{p}}_x{:}{=}e^{-2\pi {\bar{\mathsf {b}}} x}. \end{aligned}$$

### Theorem 2

Let $(\zeta ,\sigma ,E,{\bar{E}})\in \mathbb {C}^4$ be such that

119 $$\begin{aligned} {\check{f}}(p_\sigma )=\mathsf {q} p_{\zeta +\sigma }\check{f}\Big (\frac{p_\sigma }{\mathsf {q}^2}\Big ),\quad \check{\bar{f}}(\bar{p}_\sigma )=\bar{\mathsf {q}}\bar{p}_{\zeta +\sigma }\check{\bar{f}}\Big (\frac{\bar{p}_\sigma }{\bar{\mathsf {q}}^2}\Big ),\quad \Xi (\sigma )=\Xi (\zeta -\sigma ), \end{aligned}$$

where

120 $$\begin{aligned} f\in V_{\mathsf {q},\mathsf {q}p_\zeta ,E}\setminus \{0\}, \quad \bar{f}\in V_{\bar{\mathsf {q}},\bar{\mathsf {q}}{\bar{p}}_\zeta ,\bar{E}}\setminus \{0\},\quad {\check{f}}{:}{=}\frac{f}{\phi _{\mathsf {q},E}}, \quad \check{{\bar{f}}}{:}{=}\frac{\bar{f}}{\phi _{\bar{\mathsf {q}},{\bar{E}}}} \end{aligned}$$

and

121 $$\begin{aligned} \Xi (t){:}{=}-e^{\pi it(t+2\zeta )}\frac{{\check{f}}(p_t)\bar{p}_t}{\check{\bar{ f}}(\bar{ p}_t) p_t}. \end{aligned}$$

Then, the function

122 $$\begin{aligned} \Psi (x)=e^{2\pi ic_\mathsf {b}x}\frac{e^{\pi i x^2}f(p_x)\phi _{\bar{\mathsf {q}},\bar{ E}}({\bar{p}}_x)+\frac{p_x}{\bar{p}_x}\Xi (\sigma ) e^{-2\pi i\zeta x}\bar{f}(\bar{p}_x)\phi _{{\mathsf {q}},E}(p_x)}{\vartheta (p_{x-\sigma }; {\mathsf {q}}^2)\vartheta (p_{x+\sigma -\zeta };{\mathsf {q}}^2)} \end{aligned}$$

is a holomorphic solution of the system of difference Eqs. (55), (56).

### Proof

Based on two possible asymptotics at $x\rightarrow +\infty$, the most general ansatz for the common eigenfunction of our spectral problem is of the form

123 $$\begin{aligned} \Psi (x)=\Psi _1(x)+\Xi '\Psi _2(x), \end{aligned}$$

where

124 $$\begin{aligned}&\Psi _1(x){:}{=}\psi _1(x) {\tilde{h}}(p_x)\phi _{\bar{\mathsf {q}},\bar{E}}(\bar{ p}_x),\quad h\in F_ {\mathsf {q},E}\setminus \mathbb {C}\phi _{\mathsf {q},E}, \end{aligned}$$

125 $$\begin{aligned}&\Psi _2(x){:}{=}\psi _2(x) \phi _{\mathsf {q},E}(p_x)\tilde{\bar{h}}(\bar{p}_x), \quad \bar{ h}\in F_{\bar{\mathsf {q}},\bar{ E}}\setminus \mathbb {C}\phi _{\bar{\mathsf {q}},\bar{ E}}, \end{aligned}$$

and $$\Xi '\in \mathbb {C}$$. $$\square $$

### Remark 9

If the bar operation is the complex conjugation, the two functions are related as follows

126 $$\begin{aligned} \overline{\Psi _1(x)}=e^{\pi i x^2}\Psi _2(x),\quad \forall x\in \mathbb {R}. \end{aligned}$$

That implies that if $|\Xi '|=1$, then

127 $$\begin{aligned} \Xi '\overline{\Psi (x)}=e^{\pi i x^2}\Psi (x),\quad \forall x\in \mathbb {R}. \end{aligned}$$

An important additional condition for this ansatz, to be called *Requirement(I)*, is that the functions $\Psi _1(x)$ and $\Psi _2(x)$ should share one and the same set of poles.

If one chooses nonzero $h=f g$ and $\bar{ h}=\bar{ f }\bar{ g}$, where

128 $$\begin{aligned} (f,g)\in V_{{\mathsf {q}},\alpha ,E}\times T^1_{{\mathsf {q}}^2,{\mathsf {q}}^2\alpha }\ \text { and }\ (\bar{ f},\bar{g})\in V_{\bar{\mathsf {q}},{\bar{\alpha }},{\bar{E}}}\times T^1_{\bar{\mathsf {q}}^2,\bar{\mathsf {q}}^2{\bar{\alpha }}} \end{aligned}$$

for some $\alpha ,{\bar{\alpha }}\in \mathbb {C}_{\ne 0}$, then the denominators simplify to $[\phi _{\mathsf {q},E},f]$ and $[\phi _{\bar{\mathsf {q}},{\bar{E}}},{\bar{f}}]$ [(see eq. (110)] to become elements of 2-dimensional vector spaces $T^2_{\mathsf {q}^2,\mathsf {q}/\alpha }$ and $T^2_{\bar{\mathsf {q}}^2,\bar{\mathsf {q}}/{\bar{\alpha }}}$, respectively. By adjusting the normalisations of *f* and ${\bar{f}}$, we can assume that

129 $$\begin{aligned}&[\phi _{{\mathsf {q}},E},f](z)=\vartheta \Big (\frac{z}{s};{\mathsf {q}}^2\Big )\vartheta \Big (\frac{zs{\mathsf {q}}}{\alpha };{\mathsf {q}}^2\Big ),\nonumber \\&[\phi _{\bar{\mathsf {q}},\bar{ E}},\bar{ f}](z)=\vartheta \Big (\frac{z}{\bar{ s}};\bar{\mathsf {q}}^2\Big )\vartheta \Big (\frac{z\bar{ s} \bar{\mathsf {q}}}{{\bar{\alpha }}};\bar{\mathsf {q}}^2\Big ), \end{aligned}$$

where

130 $$\begin{aligned} s=s(E),\quad \bar{ s}=\bar{ s}(\bar{ E}) \end{aligned}$$

are some chosen zeros of $[\phi _{\mathsf {q},E},f]$ and $[\phi _{\bar{\mathsf {q}},\bar{ E}},\bar{ f}]$, respectively.

### Remark 10

By solving Eq. (130) for $E=E(s)$ and $\bar{E}=\bar{ E}(\bar{ s})$, one can think of *s* and $\bar{ s}$ as independent variables.

In order to fulfil Requirement(I), to rewrite $[\phi _{\mathsf {q},E},f](z)$ by using the substitutions

131 $$\begin{aligned} z\mapsto p_x ,\quad \alpha \mapsto {\mathsf {q}}p_\zeta ,\quad s\mapsto p_\sigma \end{aligned}$$

and the modularity property (27)

132 $$\begin{aligned}&[\phi _{{\mathsf {q}},E},f](z)\mapsto \vartheta (p_{x-\sigma };{\mathsf {q}}^2)\vartheta (p_{x+\sigma -\zeta };{\mathsf {q}}^2)\nonumber \\&\quad = \vartheta ({\bar{p}}_{x-\sigma };\bar{\mathsf {q}}^2)\vartheta (\bar{p}_{x+\sigma -\zeta };\bar{\mathsf {q}}^2)e^{2\pi i((x-\sin \theta -\frac{1}{2}\zeta )^2+(\sigma -\frac{1}{2}\zeta )^2)}\frac{i}{\mathsf {b}^{2}}. \end{aligned}$$

Thus, Requirement(I) is fulfilled if we substitute in $[\phi _{\bar{\mathsf {q}},\bar{ E}},\bar{ f}]$

133 $$\begin{aligned} {\bar{\alpha }}\mapsto \bar{\mathsf {q}}\bar{ p}_\zeta ,\quad \bar{ s}\mapsto \bar{ p}_\sigma . \end{aligned}$$

In this way, the first two relations in (119) become equivalent to (130).

Putting everything together, we obtain

134 $$\begin{aligned} \Psi (x)=e^{2\pi ic_\mathsf {b}x}\frac{e^{\pi i x^2}f(z)\phi _{\bar{\mathsf {q}},\bar{ E}}(\bar{ z})+\Xi e^{-2\pi i(\zeta +2\sin \theta )x}\bar{ f}(\bar{z})\phi _{\mathsf {q},E}(z)}{\vartheta (\frac{z}{s};{\mathsf {q}}^2)\vartheta (\frac{zs {\mathsf {q}}}{\alpha };{\mathsf {q}}^2)} \end{aligned}$$

with substitutions (131), (133) and $\bar{ z}\mapsto \bar{ p}_x$, and the parameter

135 $$\begin{aligned} \Xi {:}{=}\Xi 'ie^{2\pi i((\sin \theta +\zeta /2)^2+(\sigma -\zeta /2)^2)-2i\theta } \end{aligned}$$

that we choose by the condition of cancellation of the pole of $\Psi (x)$ at $x=\sigma$ with the result

136 $$\begin{aligned} \Xi =\Xi (\sigma ){:}{=}-e^{\pi i\sigma (\sigma +2\zeta )}\frac{\check{f}(s)\bar{ s}}{\check{\bar{ f}}(\bar{ s}) s} \end{aligned}$$

equivalent to (121) under the substitutions $s\mapsto p_\sigma$ and $\bar{ s}\mapsto \bar{ p}_\sigma$.

### Remark 11

Equality (136) implies that all the poles of $\Psi (x)$ at $\sigma +i\mathsf {b}m+i\mathsf {b}n$, $m,n\in \mathbb {Z}$, are cancelled as well. The proof is based on the relations (114) and their complex conjugate counterparts.

Now, the last step in the proof is to fulfil *Requirement(II)* which consists of cancelling the remaining poles of $\Psi (x)$. Due to the equivalence (117) and Remark 11, Requirement(II) boils down to a single equation

137 $$\begin{aligned} \Xi (\sigma )=\Xi (\zeta -\sigma ). \end{aligned}$$

which determines a discrete set of solutions for the variable $\sigma$, while the corresponding eigenvalues of the Hamiltonian are given through the implicit function $E=E(\sigma )$.

In conclusion, we would like to remark that the variable $\zeta$ in Theorem 2 is an auxiliary parameter whose role is unclear to us. We conjecture that the eigenvalues of the Hamiltonian as well as the eigenvectors are independent of $\zeta$. This is confirmed by numerical calculations.

## Footnotes

1 By inclusion of operators $S\subset T$, we mean that ${\mathcal {D}}(S)\subset {\mathcal {D}}(T)$ and $S=T\vert _{{\mathcal {D}}(S)}$.

## References

[1] Aganagic M, Cheng MCN, Dijkgraaf R, Krefl D, Vafa C (2012). Quantum geometry of refined topological strings. J. High Energy Phys..

[2] Aganagic M, Dijkgraaf R, Klemm A, Mariño M, Vafa C (2006). Topological strings and integrable hierarchies. Comm. Math. Phys..

[3] Babelon O, Kozlowski KK, Pasquier V (2018). Baxter operator and Baxter equation for $$q$$-Toda and $${\rm Toda}_2$$ chains. Rev. Math. Phys..

[4] V. V. Bazhanov, S. L. Lukyanov, and A. B. Zamolodchikov. Spectral determinants for Schrödinger equation and $${\bf Q}$$-operators of conformal field theory. In: Proceedings of the Baxter Revolution in Mathematical Physics (Canberra, 2000), volume 102, pages 567–576, 2001

[5] Codesido S, Grassi A, Mariño M (2017). Spectral theory and mirror curves of higher genus. Ann. Henri Poincaré.

[6] Dorey P, Tateo R (1999). Anharmonic oscillators, the thermodynamic Bethe ansatz and nonlinear integral equations. J. Phys. A.

[7] Faddeev LD (1995). Discrete Heisenberg-Weyl group and modular group. Lett. Math. Phys..

[8] Grassi A, Hatsuda Y, Mariño M (2016). Topological strings from quantum mechanics. Ann. Henri Poincaré.

[9] Grassi, A., and Mariño, M. .: The complex side of the TS/ST correspondence. arXiv:1708.08642, (2017)

[10] Hatsuda Y, Mariño M, Moriyama S, Okuyama K (2014). Non-perturbative effects and the refined topological string. J. High Energy Phys..

[11] Hatsuda Y, Moriyama S, Okuyama K (2013). Instanton effects in ABJM theory from Fermi gas approach. J. High Energy Phys..

[12] Huang M-X, Wang X-F (2014). Topological strings and quantum spectral problems. J. High Energy Phys..

[13] Källén J, Mariño M (2016). Instanton effects and quantum spectral curves. Ann. Henri Poincaré.

[14] Kashaev, R.M., and Sergeev, S.M.: Spectral equations for the modular oscillator. Rev. Math. Phys., 30(7):1840009, 28, (2018). Preprint arXiv:1703.06016

[15] Kashani-Poor, A.-K.: Quantization condition from exact WKB for difference equations. arXiv:1604.01690, (2016)

[16] Mariño, M.: Spectral theory and mirror symmetry. In: String-Math 2016 of Proc. Sympos. Pure Math, vol. 98, pp. 259–294. Amer. Math. Soc., Providence, RI, (2018)

[17] Mariño M, Putrov P (2012). ABJM theory as a Fermi gas. J. Stat. Mech. Theory Exp..

[18] Mariño, M., and Zakany, S.: Exact eigenfunctions and the open topological string. arXiv:1606.05297, (2016)

[19] Mironov A, Morosov A (2010). Nekrasov functions and exact Bohr-Sommerfeld integrals. J. High Energy Phys..

[20] Nekrasov, N.A., and Shatashvili, S.L.: Quantization of integrable systems and four dimensional gauge theories. In: XVIth International Congress on Mathematical Physics, pp. 265–289. World Sci. Publ., Hackensack, NJ, (2010)

[21] Schmüdgen K (2012). Unbounded self-adjoint operators on Hilbert space.

[22] Sergeev SM (2005). A quantization scheme for modular $$q$$-difference equations. Teoret. Mat. Fiz..

